# 802. *Corynebacterium striatum* Outbreak Among Ventilated COVID-19 Patients in an Acute Care Hospital – California, 2021

**DOI:** 10.1093/ofid/ofab466.998

**Published:** 2021-12-04

**Authors:** Janice J Kim, Nancy E Turner, Emily Holman, Linda Lefrak, Fady A Youssef, Patrizia Richardson, Rituparna Mukhopadhyay, John Crandall, Henry Su, Erin Epson

**Affiliations:** 1 California Department of Public Health, Richmond, California; 2 Memorialcare Long Beach Medical Center, Simi Valley, California; 3 Long Beach Department of Health and Human Services, Long Beach, California; 4 Long Beach Memorial Medical Center, Long Beach, California; 5 Long Beach Memorial Medical Center, Redondo Beach, California; 6 MemorialCare Long Beach Medical Center, Long Beach, California

## Abstract

**Background:**

*Corynebacterium striatum* (CS), a common human commensal colonizing the skin and nasopharynx, has been associated with nosocomial infections in immunocompromised and chronically ill patients. During the winter 2020-2021 COVID-19 surge, a 420-bed California hospital reported a marked increase in CS respiratory cultures among ventilated COVID-19 patients. We conducted a public health investigation to assess and mitigate nosocomial transmission and contributing infection prevention and control (IPC) practices.

**Methods:**

A case was defined as a patient with CS in respiratory cultures from January 1, 2020 - February 28, 2021. We reviewed clinical characteristics on a subset of cases in 2021 and IPC practices in affected hospital locations. CS respiratory isolates collected on different dates and locations were assessed for relatedness by whole genome sequencing (WGS) on MiSeq.

**Results:**

Eighty-three cases were identified, including 75 among COVID-19 patients (Figure 1). Among 62 patients identified in 2021, all were ventilated; 58 also had COVID-19, including 4 cases identified on point prevalence survey (PPS). The median time from admission to CS culture was 19 days (range, 0-60). Patients were critically ill; often it was unclear whether CS cultures represented colonization or infection. During the COVID-19 surge, two hospital wings (7W and 7S) were converted to negative-pressure COVID-19 units. Staff donned and doffed personal protective equipment in anterooms outside the units; extended use of gowns was practiced, and lapses in glove changes and hand hygiene (HH) between patients likely occurred. In response to the CS outbreak, patients were placed in Contact precautions and cohorted. Staff were re-educated on IPC for COVID-19 patients. Gowns were changed between CS patients. Subsequent PPS were negative. Two CS clusters were identified by WGS: cluster 1 (5 cases) in unit 7W, and cluster 2 (2 cases) in unit 7S (Figure 2).

Figure 1. Corynebacterium striatum Respiratory Cultures January 2020-February 2021

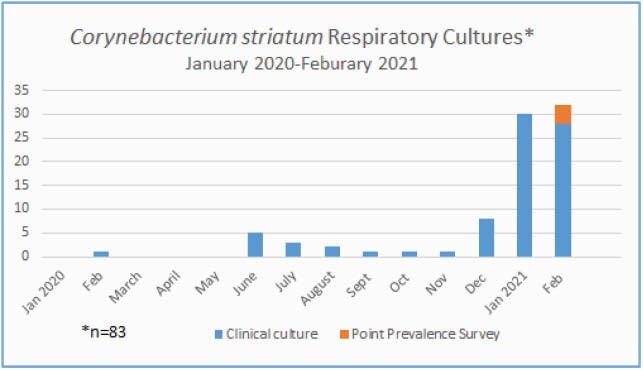

Figure 2. Phylogenetic Tree Corynebacterium striatum Isolates

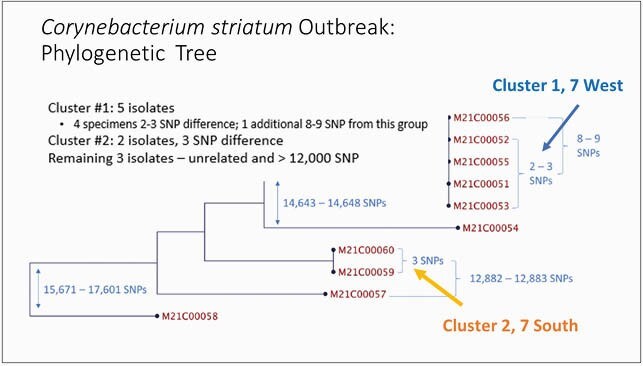

**Conclusion:**

A surge in patients, extended use of gowns and lapses in core IPC practices including HH and environmental cleaning and disinfection during the winter 2020-2021 COVID-19 surge likely contributed to this CS outbreak. WGS provides supportive evidence for nosocomial CS transmission among critically ill COVID-19 patients.

**Disclosures:**

**All Authors**: No reported disclosures

